# Nursing Workflow Change in a COVID-19 Inpatient Unit Following the Deployment of Inpatient Telehealth: Observational Study Using a Real-Time Locating System

**DOI:** 10.2196/36882

**Published:** 2022-06-17

**Authors:** Stacie Vilendrer, Mary E Lough, Donn W Garvert, Monique H Lambert, Jonathan Hsijing Lu, Birju Patel, Nigam H Shah, Michelle Y Williams, Samantha M R Kling

**Affiliations:** 1 Evaluation Sciences Unit, Division of Primary Care and Population Health Department of Medicine Stanford University School of Medicine Stanford, CA United States; 2 Office of Research Patient Care Services Stanford Health Care Palo Alto, CA United States; 3 Center for Biomedical Informatics Research Stanford University School of Medicine Stanford, CA United States

**Keywords:** telemedicine, telehealth, informatics, real-time locating system, COVID-19, pandemic, nursing, patient safety, PPE, virtual care, nurses, patient outcomes, pathogen exposure, health risk, health care staff, health care professional

## Abstract

**Background:**

The COVID-19 pandemic prompted widespread implementation of telehealth, including in the inpatient setting, with the goals to reduce potential pathogen exposure events and personal protective equipment (PPE) utilization. Nursing workflow adaptations in these novel environments are of particular interest given the association between nursing time at the bedside and patient safety. Understanding the frequency and duration of nurse-patient encounters following the introduction of a novel telehealth platform in the context of COVID-19 may therefore provide insight into downstream impacts on patient safety, pathogen exposure, and PPE utilization.

**Objective:**

The aim of this study was to evaluate changes in nursing workflow relative to prepandemic levels using a real-time locating system (RTLS) following the deployment of inpatient telehealth on a COVID-19 unit.

**Methods:**

In March 2020, telehealth was installed in patient rooms in a COVID-19 unit and on movable carts in 3 comparison units. The existing RTLS captured nurse movement during 1 pre- and 5 postpandemic stages (January-December 2020). Change in direct nurse-patient encounters, time spent in patient rooms per encounter, and total time spent with patients per shift relative to baseline were calculated. Generalized linear models assessed difference-in-differences in outcomes between COVID-19 and comparison units. Telehealth adoption was captured and reported at the unit level.

**Results:**

Change in frequency of encounters and time spent per encounter from baseline differed between the COVID-19 and comparison units at all stages of the pandemic (all *P*<.001). Frequency of encounters decreased (difference-in-differences range –6.6 to –14.1 encounters) and duration of encounters increased (difference-in-differences range 1.8 to 6.2 minutes) from baseline to a greater extent in the COVID-19 units relative to the comparison units. At most stages of the pandemic, the change in total time nurses spent in patient rooms per patient per shift from baseline did not differ between the COVID-19 and comparison units (all *P*>.17). The primary COVID-19 unit quickly adopted telehealth technology during the observation period, initiating 15,088 encounters that averaged 6.6 minutes (SD 13.6) each.

**Conclusions:**

RTLS movement data suggest that total nursing time at the bedside remained unchanged following the deployment of inpatient telehealth in a COVID-19 unit. Compared to other units with shared mobile telehealth units, the frequency of nurse-patient in-person encounters decreased and the duration lengthened on a COVID-19 unit with in-room telehealth availability, indicating “batched” redistribution of work to maintain total time at bedside relative to prepandemic periods. The simultaneous adoption of telehealth suggests that virtual care was a complement to, rather than a replacement for, in-person care. However, study limitations preclude our ability to draw a causal link between nursing workflow change and telehealth adoption. Thus, further evaluation is needed to determine potential downstream implications on disease transmission, PPE utilization, and patient safety.

## Introduction

The COVID-19 pandemic prompted the widespread implementation of telehealth throughout the health care sector to protect patients and health care workers by reducing the risk of infection [1,2]. In the inpatient setting, telehealth was previously used to connect hospitalized patients in rural settings with remote specialists [3], but has recently expanded to facilitate digital communication between patients and on-site clinicians [4-6]. The impact of inpatient telehealth on infection reduction [7], clinical workflows [8,9], patient safety [10], and personal protective equipment (PPE) utilization [10,11] is still under investigation. In the context of a pandemic, telehealth’s impact on infection control and clinical care is of particular interest, although existing evaluations have relied on PPE inventory data and patient and provider satisfaction surveys [10,11]. However, the expanding use of real-time locator systems (RTLSs), as an objective source of location and time data, in inpatient settings offers a unique opportunity to understand how clinical workflows adapt to these novel circumstances.

An RTLS captures the time spent in specific locations, providing a direct measure of workflow for health care professionals as well as a proxy for outcomes associated with staff movement, such the use of PPE upon each entrance into a room [12-17]. In the context of infection control, RTLS data have been used to identify possible transmission: one study demonstrated higher sensitivity and specificity using RTLS-based contact tracing than an audit log capturing electronic health record (EHR) logins at diverse workstations [7]. Availability of an RTLS provides an opportunity to evaluate the impact of new technologies or clinical circumstances (eg, infectious outbreak) on clinical workflows. A recent study analyzing RTLS data in the emergency setting provided an analytical framework to understand possible clinician workflow adaptations, although no change was ultimately detected in this setting [9].

The impact of telehealth on nursing workflows is of particular interest, as nurses spend approximately 6-fold more time at the bedsides of hospitalized patients than attending physicians [18]. More time in direct nurse-patient encounters has been associated with improved patient safety [19] and satisfaction [17]. The manner in which nurses structure in-person encounters with patients depends on local hospital guidelines [19], but can also vary between individuals, according to the level of training, and time of day [20]. Bedside encounters for inpatients under isolation precautions (such as is required during COVID-19 treatment) appear to be reduced relative to other inpatients [21]. This reduction may contribute to isolated patients receiving substandard care [22-24]*.* Understanding direct nurse-patient care following the introduction of a novel telehealth platform in the context of COVID-19 may provide insight into the downstream impacts on patient safety, pathogen exposure, and PPE utilization.

Thus, we aimed to evaluate changes in nursing workflow relative to prepandemic levels using an RTLS following the deployment of inpatient telehealth on a COVID-19 unit. Given isolation precautions and ready availability of telehealth equipment on the COVID-19 unit, we hypothesized a reduction in the frequency and duration of in-person nurse-patient encounters in the COVID-19 unit relative to prepandemic comparator units.

## Methods

### Design

Telehealth was implemented throughout an acute care academic hospital in response to the COVID-19 pandemic in March of 2020. The setup of telehealth differed between the hospital’s primary COVID-19 unit and comparison units. Our primary aim was to explore changes in nursing workflows in these novel circumstances through a retrospective, observational evaluation using RTLS data to capture the frequency of direct nurse-patient encounters at the bedside, time nurses spent in patient rooms per encounter, and total time nurses spent with each patient in the patient room per shift. For each outcome, the change from the prepandemic level was calculated, and difference-in-differences analyses were used to determine if changes in nurse movement differed between the COVID-19 unit and comparison units. The simultaneous adoption of telehealth in terms of video call frequency and duration was captured and is reported at the unit level.

### Setting

The academic acute care hospital is in a major metropolitan area in the western United States and serves a diverse population. The four inpatient units in this evaluation were identical in size and layout with 22-bed single-room capacity. Prepandemic, these four units focused on inpatient general medicine populations. Other units serving primarily surgical, oncological, and intensive care patients were excluded. At the beginning of the pandemic, one of the units became the primary COVID-19 unit for the hospital and was therefore compared to the other three units. Hospital administration reported that standard registered nurse-to-patient ratio on the four units ranged from 1:4 to 1:3, varying with disease acuity based on state law [25]. The registered nurse-to-patient ratio on the COVID-19 unit shifted from 1:4 to 1:3 by April 2020.

### Timeline

The first patient tested positive for SARS-CoV-2 in the hospital’s emergency department on March 2, 2020, and local stay-at-home orders were announced on March 16, 2020 [26]. For the purposes of this evaluation, data from all data sources were collected from January 1, 2020, through December 27, 2020. These data captured 6 stages of the pandemic that were defined based on local case rates [27] (for criteria and specific dates, see section A1 of Multimedia Appendix 1): (1) prepandemic, (2) telehealth rollout, (3) nonsurge #1, (4) surge #1, (5) nonsurge #2, and (6) surge #2.

### Telehealth Deployment

In response to the pandemic, telehealth was rapidly implemented throughout inpatient settings in mid-March 2020, as previously reported [4] and described in section A2 of Multimedia Appendix 1. In the COVID-19 inpatient unit, telehealth hardware with a video tablet was permanently installed in each patient room. However, in non-COVID units, shared telehealth video tablets were available only on mobile carts, which could be transported into a patient room as needed. A member of the clinical team was required to roll the cart into the patient room prior to each use, and these units were sometimes unavailable for patients if they were already in use by another patient.

All clinical team members, including hospitalist and specialist physicians, nurses, respiratory therapists, trainees, and clinical researchers, received instructions on and were encouraged to incorporate telehealth into their clinical or research activities. Patients received incoming calls passively given the default automatic turn-on feature of the video tablet, but received no other instruction or guidance on its use [28].

### Data Sources and Processing

#### RTLS Nurse Movement Data

The primary data source for this evaluation was extracted from the existing RTLS platform (Midmark, Dayton, OH). The RTLS captured movement of nurses into and out of patient rooms on the selected units. These data were used to calculate the following 3 outcomes related to nurses’ movement and direct patient care: (1) number of nurse-patient direct encounters within patient rooms, (2) time nurses spent in patient room per encounter, and (3) total time nurses spent in the patient room with each patient per shift.

The key components of the RTLS are infrared and radiofrequency sensors installed in each room and staff badges worn alongside their name badge [18,29]. Line of sight between the room sensor and staff member’s badge triggered the system to record an event such as entry of a nurse into a patient room. The badge emitted a ping every 1 to 3 seconds to indicate presence in the room. Only events longer than 5 seconds were recorded in the system. At installation, sensitivity settings were optimized based on the geometric configuration and construction materials, but were not reassessed for this evaluation [9,18].

Since the direct line of sight between the room sensor and nurse badge could be briefly interrupted (eg, by turning away from the sensor), a single nurse-patient encounter could appear as several short, consecutive events. Thus, to identify unique direct nurse-patient encounters and calculate the time nurses spent in a patient room, multiple successive RTLS events that occurred within 30 seconds of another and were associated with an individual nurse in a single patient room were collapsed into a single direct nurse-patient encounter. The duration of individual encounters was summed to calculate the total time nurses spent with each patient per shift using the first and last timestamp of each encounter associated with a unique nurse in a specific patient room. Since nursing needs and thus workflows may differ between shifts [20], movement from 7:00 AM to 6:59 PM (morning shift) is presented separately from movement from 7:00 PM to 6:59 AM (night shift).

Staff RTLS badges were linked within the system to an employee identifier and role. Nurses categorized as “nurse” or “float nurse” within the RTLS were included in the analysis. Hospital administration and managers encourage all nurses to wear the badges to utilize the system’s beneficial features, including automatic silencing of patient room alarms when a nurse enters a room and a discreet button to call security. Compliance with badge wearing is near universal among nursing staff compared to other members of the clinical team (eg, physicians) [9]. However, the evaluation team could not and did not confirm that all nurses were compliant during the observation period. The process to access these data is briefly described in section A3 of Multimedia Appendix 1.

#### EHR Patient Data

Data were extracted from the EHR (Epic, Verona, WI, USA) to determine the presence of a patient in each hospital room in the unit at midnight, and the results of the patient’s most recent COVID-19 test result (positive or negative) within the prior 14 days or from hospital admission. Besides this information, no other patient-level data such as identifiers or clinical characteristics were obtained. The RTLS and EHR data were then merged by patient room and time to identify direct encounters between nurses and patients.

#### Telehealth Utilization Data

Data were extracted from Zoom video conferencing software and included the unit associated with the host (originating hardware) user ID, call start time, call end time, and number of participants. Only calls lasting between 30 seconds and 2 hours with 2 or more participants were included in analysis. Patients were not instructed or encouraged to initiate calls to clinical staff or family members themselves during the observation period, although anecdotal reports suggest that nurses occasionally set up calls between patients and their families. This data platform did not link a telehealth encounter to individual physicians, staff members (nurses), or patients. Consequently, telehealth use described all possible use cases at the unit level.

#### Data Analysis

Descriptive statistics were generated to describe patient census by COVID-19 status and telehealth utilization in the COVID-19 and comparison units prepandemic during the 5 stages of the pandemic. Telehealth utilization is expressed as the number of telehealth calls per patient (using the midnight patient census) per unit for all case uses such as clinical encounters, research activities, and patient-family connections. Since telehealth events could not be linked to individual users (patients or health care workers), no additional analysis was performed with these data.

To investigate differences in the three primary outcomes based on RTLS data between the COVID-19 and comparison units, a difference-in-differences approach was applied. Change in each outcome was calculated for each of the 5 stages of the pandemic, as defined by local case rates, relative to prepandemic levels. To determine if the change from the prepandemic stage differed between the COVID-19 and comparison units, difference-in-differences was determined using a generalized linear model in SAS (version 9.4, SAS Institute Inc) for each of the three outcomes.

Means, standard deviations, and ranges are reported where appropriate. For all models, *P*<.05 was considered statistically significant. When multiple comparisons were made, *P* values were adjusted using an adaptive, two-stage linear setup procedure to control the false discovery rate [30].

### Ethics Considerations

Data were obtained from multiple sources, including RTLS movement data at the individual nurse level, EHR data at the patient and thus room level, and telehealth log data at the unit level. All data were deidentified and data points that did not occur in patient rooms were removed before being sent to the analytics team to protect the anonymity of the workforce. The evaluation was exempt per the Stanford University's institutional review board (protocol 55927).

## Results

### Overview

The COVID-19 unit was the main care location for hospitalized patients diagnosed with COVID-19, but it was also the care location for some patients without COVID-19 (see Table S1 in Multimedia Appendix 2). In contrast, the comparison units cared for very few patients diagnosed with COVID-19 prior to the final evaluation stage, surge #2, when the number of COVID-19 patients increased throughout the hospital.

**Figure 1:**
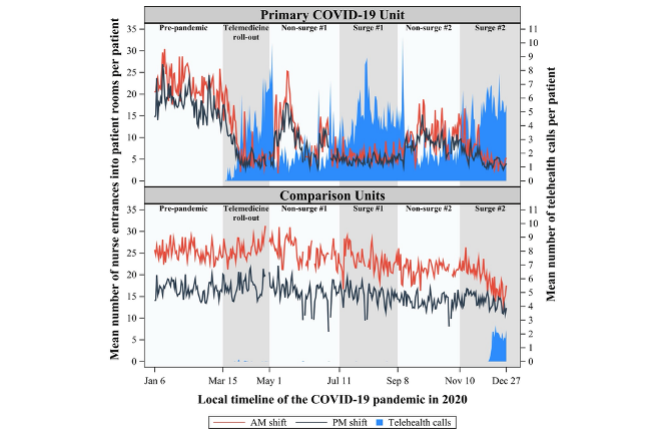
Daily mean number of times nurses entered patient rooms by shift on a COVID-19 unit and three comparison units during a telehealth implementation in the context of the SARS-CoV-2 pandemic.

**Figure 2:**
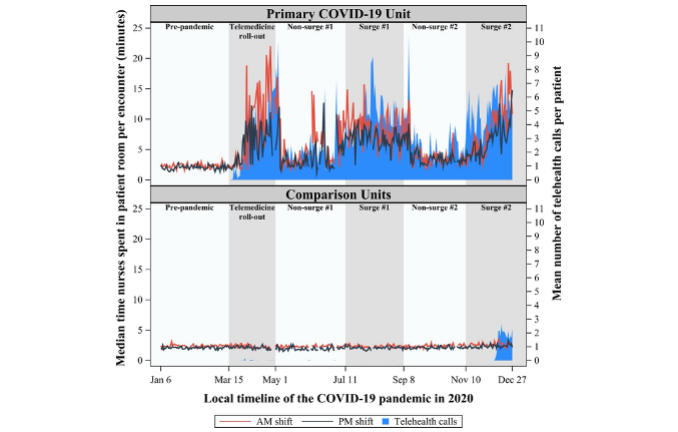
Median time (minutes) nurses spent in patient rooms per encounter by shift on a primary COVID-19 unit and three comparison units during a telehealth implementation in the context of the SARS-CoV-2 pandemic.

**Figure 3:**
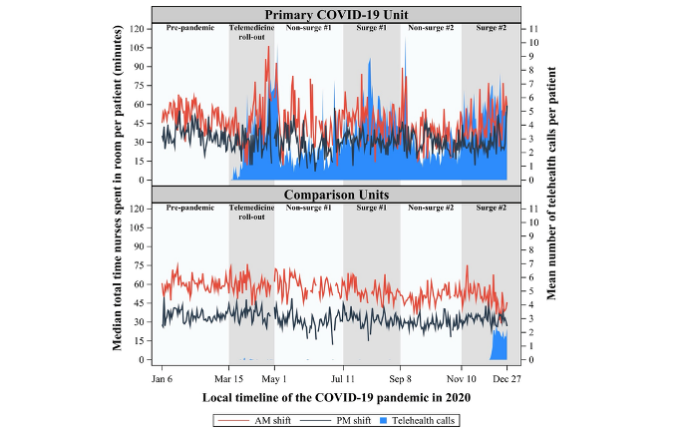
Median total time (minutes) nurses spent in patient rooms per patient per shift on a primary COVID-19 unit and three comparison units during a telehealth implementation in the context of the SARS-CoV-2 pandemic.

The comparison units demonstrated little adoption of telehealth throughout most of the pandemic, whereas the primary COVID-19 unit quickly adopted this technology (Figures 1-3), initiating 15,088 inpatient telehealth video encounters, resulting in a cumulative duration of 1660 hours throughout the observation period. On average, encounters were 6.6 (SD 13.6) minutes per telehealth call.

Results are presented in order of the three primary outcomes, including the change in the frequency nurses entered patient rooms, time nurses spent in patient rooms per entry, and total time nurses spent in each patient room per shift.

### Direct Nurse-Patient Encounters

Across both the COVID-19 and comparison units, 876,177 unique direct nurse-patient encounters were identified, including 226,326 encounters prepandemic and 649,791 during the pandemic. Of these, 18,011 encounters were linked to a patient who was positive for COVID-19.

The daily mean number of nurse entries in patient rooms by shift (morning and night shifts) for the primary COVID-19 unit and comparison units is shown in Figure 1. In the prepandemic stage, nurses in the primary COVID-19 unit entered patient rooms less frequently than nurses in the comparison units for the morning and more frequently for the night shift (Table 1). Because the COVID-19 versus comparator units differed in the number of prepandemic nurse-patient encounters, a difference-in-differences analysis was applied to determine if change in this outcome relative to baseline differed between the COVID-19 and comparison units.

For each stage of the pandemic, change in number of times nurses entered patient rooms relative to that prepandemic are shown in Table 1 for the COVID-19 unit and comparison units. In the COVID-19 unit, the decrease in encounters relative to prepandemic levels ranged from –9.2 to –16.8 nurse-patient encounters per shift, whereas in the comparison units, these fluctuations were less pronounced (range –5.9 to +0.8 nurse-patient encounters per shift). At all stages of the pandemic, for both morning and night shifts, the number of times nurses entered patient rooms decreased from prepandemic levels to a greater extent for nurses on the COVID-19 unit than for nurses in the comparison units (all *P*<.001). Nurses in the COVID-19 unit entered patient rooms less frequently during each surge period when compared to the nonsurge period just before; this pattern was seen for both the morning and night shifts (all *P*<.001; estimates range from 3.4 to 4.8 additional entries).

**Table 1:** Number of direct nurse-patient encounters per shift, difference in number of encounters from prepandemic stage, and difference-in-differences between a primary COVID-19 unit and three comparison units in the context of the SARS-CoV-2 pandemic.

Stage			Primary COVID-19 unit				Comparison units				Difference in differences		*P* value^a^
			Number of direct nurse-patient encounters, mean (SD)		Difference from prepandemic		Number of direct nurse-patient encounters, mean (SD)		Difference from prepandemic				
**Prepandemic baseline**													
	AM shift	22.4 (3.2)		N/A^b^		25.3 (1.8)		N/A		N/A		N/A	
	PM shift	17.8 (3.1)		N/A		16.6 (1.7)		N/A		N/A		N/A	
**Telehealth rollout**													
	AM shift	8.9 (5.7)		–13.5		25.8 (2.0)		0.5		–14.1		<.001	
	PM shift	6.8 (3.9)		–11.0		17.3 (2.1)		0.8		–11.8		<.001	
**Nonsurge #1**													
	AM shift	10.5 (5.5)		–11.9		25.3 (2.5)		0.0		–11.9		<.001	
	PM shift	8.6 (3.8)		–9.2		15.9 (2.6)		–0.7		–8.5		<.001	
**Surge #1**													
	AM shift	5.6 (1.4)		–16.8		23.2 (2.6)		–2.1		–14.7		<.001	
	PM shift	4.7 (0.8)		–13.1		15.5 (2.0)		–1.1		–12.0		<.001	
**Nonsurge #2**													
	AM shift	10.2 (3.4)		–12.2		21.3 (1.8)		–4.0		–8.2		<.001	
	PM shift	8.5 (2.2)		–9.3		13.9 (1.6)		–2.7		–6.6		<.001	
**Surge #2**													
	AM shift	6.6 (3.1)		–15.8		19.4 (3.0)		–5.9		–9.9		<.001	
	PM shift	5.1 (1.8)		–12.7		14.0 (1.6)		–2.6		–10.1		<.001	

^a^Difference-in-differences was statistically tested using a generalized linear model.

^b^N/A: not applicable.

### Duration of Nurse-Patient Encounters

The daily median time nurses spent in patient rooms per shift (morning and night) for the primary COVID-19 unit and comparison units is shown in Figure 2. The downward trend in encounters in patient rooms in the COVID-19 unit (Figure 1, Table 1) corresponded with an upward shift in the amount of time nurses spent in the patient rooms per entry (Figure 2), and this pattern remained consistent throughout the pandemic.

Time nurses spent in patient rooms per entry did not differ between the COVID-19 unit and comparison units during the prepandemic stages for both the morning (*P*=.79) and night (*P*=.81) shifts (Table 2). For each stage of the pandemic, change in time nurses spent in patient rooms per encounter relative to prepandemic is shown in Table 2 for the primary COVID-19 unit and comparison units. In the COVID-19 unit, the duration of encounters increased in all phases of the pandemic, although at different rates, with mean increases of 1.8 to 6.2 minutes compared to prepandemic levels (Table 2). In the comparison units, these fluctuations were less pronounced, with no change during several phases and a range of –0.2 to 0.2 minutes per encounter (Table 2). At all stages of the pandemic, for both the morning and night shifts, the duration of nurse-patient encounters increased from prepandemic levels to a greater extent for nurses in the COVID-19 unit than for nurses in the comparison units (all *P*<.001). Nurses in the COVID-19 unit spent more time in patient rooms per entry during each surge period when compared to that in the nonsurge period just before; this pattern was seen for both the morning and night shifts (all *P*<.001; estimates range from 2.6 to 4.1 fewer minutes per entry).

**Table 2:** Duration (minutes) each nurse spent in patient rooms per encounter by shift, difference in duration of encounters, and difference-in-differences between the primary COVID-19 unit and three comparison units during a telehealth implementation in the context of the SARS-CoV-2 pandemic.

Period			Primary COVID-19 unit				Comparison units				Differences in differences		*P* value^a^
			Duration (minutes) nurse spent in patient room per encounter, mean^b^ (SD)		Difference from prepandemic		Duration (minutes) nurse spent in patient room per encounter, mean^b^ (SD)		Difference from prepandemic				
**Prepandemic baseline**													
	AM shift	2.3 (0.3)		N/A^c^		2.4 (0.2)		N/A		N/A		N/A	
	PM shift	2.0 (0.4)		N/A		2.1 (0.2)		N/A		N/A		N/A	
**Telehealth rollout**													
	AM shift	8.5 (6.3)		6.2		2.3 (0.3)		–0.1		6.2		<.001	
	PM shift	5.7 (3.0)		3.7		2.1 (0.2)		0.0		3.7		<.001	
**Nonsurge #1**													
	AM shift	5.5 (3.6)		3.2		2.3 (0.2)		–0.1		3.3		<.001	
	PM shift	4.0 (2.5)		2.0		1.9 (0.3)		–0.2		2.1		<.001	
**Surge #1**													
	AM shift	8.5 (2.6)		6.2		2.3 (0.2)		0.0		6.2		<.001	
	PM shift	6.8 (1.5)		4.8		2.1 (0.2)		0.0		4.8		<.001	
**Nonsurge #2**													
	AM shift	4.1 (2.2)		1.8		2.4 (0.2)		0.0		1.8		<.001	
	PM shift	3.8 (1.5)		1.8		2.1 (0.2)		0.0		1.8		<.001	
**Surge #2**													
	AM shift	8.2 (4.2)		5.9		2.6 (0.3)		0.2		5.7		<.001	
	PM shift	6.4 (2.7)		4.4		2.3 (0.3)		0.2		4.2		<.001	

^a^Difference in differences was statistically tested using a generalized linear model.

^b^To determine the duration a nurse spent in the patient rooms per encounter, the median value per shift was calculated. In this table, the mean represents the mean of these median values.

^c^N/A: not applicable.

### Total Time of Direct Nurse-Patient Care

The daily median total time nurses spent in patient rooms per patient per shift (morning and night) for the primary COVID-19 unit and comparison units is shown in Figure 3. During the prepandemic stage, nurses in the primary COVID-19 unit spent less total time per patient per shift than nurses in the comparison units during the morning shift (*P*<.001) but not the night shift (*P*=.57), as shown in Table 3.

In each stage of the pandemic, the change in the total time nurses were in a patient room per patient per shift relative to baseline is shown in Table 3 for the primary COVID-19 unit and comparison units. For the COVID-19 unit, the mean change in the total time nurses spent in patient rooms per patient per shift ranged from –2.8 to –13.0 minutes relative to the prepandemic times. In comparison units, the mean change relative to prepandemic was less pronounced and ranged from –9.9 to +1.6 minutes (Table 3). At most stages of the pandemic, change in total time in the patient room from prepandemic did not differ between the primary COVID-19 unit and comparison units (all *P*>.17), with the exception of the telehealth rollout phase.

**Table 3:** Total time (minutes) all nurses spent in a patient room by shift, difference in total time relative to baseline, and difference-in-differences between the primary COVID-19 unit and three comparison units during a telehealth implementation in the context of the SARS-CoV-2 pandemic.

Period		Primary COVID-19 unit			Comparison units			Differences in differences		*P* value^a^
		Total time (minutes) nurses spent in patient room per patient per shift, mean^b^ (SD)	Difference from prepandemic	Total time (minutes) nurse spent in patient room per patient per shift, mean^b^ (SD)		Difference from prepandemic				
**Prepandemic baseline**										
	AM shift	51.0 (9.1)	N/A^c^	60.0 (5.1)		N/A	N/A		N/A	
	PM shift	35.8 (7.6)	N/A	34.9 (4.4)		N/A	N/A		N/A	
**Telehealth rollout**										
	AM shift	48.2 (20.3)	–2.8	59.4 (7.3)		–0.6	–2.2		.48	
	PM shift	30.7 (10.1)	–5.1	36.5 (5.0)		1.6	–6.8		.04	
**Nonsurge #1**										
	AM shift	44.8 (17.1)	–6.2	58.7 (6.9)		–1.3	-4.9		.17	
	PM shift	28.9 (10.6)	–6.9	30.9 (6.3)		–4.0	–3.0		.26	
**Surge #1**										
	AM shift	46.2 (12.8)	–4.8	54.4 (6.5)		–5.7	0.8		.72	
	PM shift	31.4 (6.2)	–4.4	32.2 (5.3)		–2.7	–1.6		.50	
**Nonsurge #2**										
	AM shift	38.0 (14.4)	–13.0	50.4 (5.6)		–9.6	–3.4		.24	
	PM shift	30.4 (8.1)	–5.4	29.1 (4.3)		–5.8	0.4		.87	
**Surge #2**										
	AM shift	44.9 (13.6)	–6.1	50.1 (9.2)		–9.9	3.8		.24	
	PM shift	29.8 (8.8)	–6.0	32.5 (3.9)		–2.4	–3.7		.26	

^a^Difference-in-differences was statistically tested using a generalized linear model.

^b^To determine the total time nurses spent in patient rooms, the median value per shift was calculated. In this table, the mean represents the mean of these median values.

^c^N/A: not applicable.

## Discussion

### Principal Findings

Evaluating changes in the frequency and duration of direct nurse-patient encounters using an RTLS following an inpatient telehealth deployment during the COVID-19 pandemic was feasible and provided novel insights into nursing workflow redistribution in this setting. Relative to the prepandemic stage, nurses in a COVID-19 unit with in-room ready access to telehealth decreased the frequency of entries into patient rooms to a greater extent than that for nurses in other units with shared mobile telehealth units. Counter to our hypothesis, the average in-person encounter length increased proportionally, such that the total in-person time nurses spent with patients on the COVID-19 unit did not significantly differ from that in prepandemic comparator units. The simultaneous adoption of telehealth, presented at the unit level, suggests it was used as a complement to, rather than a replacement for, in-person care.

To put the above findings into context, the average decrease in encounters weighted by time period in the COVID-19 unit relative to other units was 11.25 and 9.13 encounters per patient per morning and night shift, respectively. Assuming full capacity on the 22-bed COVID-19 unit and PPE use for a quarter of such encounters (as isolation precautions were not required for every patient on the unit), these data suggest workflow adaptations saved approximately 785 PPE units over the course of a week. Similarly, given the increased time burden required in caring for isolated patients—approximately 4 minutes to don and 3 minutes to doff PPE outside the patient room [31]—workflow adaptations saved nurses an extra 78 and 64 minutes per morning and night shift, respectively.

The change in total time spent at patient bedside per shift from baseline did not differ significantly between the COVID-19 and comparison units, given the increased duration of each encounter. Past work suggests that this “batching” or “clustering” of bedside work includes performing physical assessments, administering medications, and delivering a food tray all in one bedside encounter [8,32]. The impact of workflow change to fewer, longer encounters on patient safety and satisfaction is an area for future research, although recent qualitative work suggests that COVID-19 patients overall did not feel their care was compromised and accepted the technology given the need for isolation precautions [28]. Further, the novel finding that total in-person nursing time at the bedside is unchanged following a telehealth deployment may be favorable, particularly given the known positive link between nursing time spent at the bedside and patient safety [19].

Notably, Figure 1 suggests an inverse relation between the mean number of direct care events and the mean number of telehealth calls during surge versus nonsurge periods. While nurses shifted their practice patterns to fewer, longer encounters during surges, it appears they also increased telehealth use before reverting back to standard practice patterns during nonsurge periods. This simultaneous adoption of telehealth on the COVID-19 unit suggests that virtual care was an additive complement to, rather than a replacement for, in-person care in terms of total time spent with the patient. Our findings therefore point to the possible role inpatient telehealth could play in improving patient safety for isolated patients. Specifically, understanding the optimal ratio for in-person to virtual encounters as well as patient and clinician triggers for each type of encounter may further inform telehealth use across varied inpatient settings.

The COVID-19 unit and comparison units had important differences that likely influenced the adoption of telehealth. In the COVID-19 unit, hardware was readily available in each patient room, whereas the comparison units had a limited number of carts in a central location on the unit; the hardware had to be retrieved and then set up in the patient rooms prior to use. In addition to the ready availability of technology, the increased threat of pathogen exposure on the COVID-19 unit likely further promoted rapid staff uptake relative to comparison units, which did not adopt telehealth until late in the observation period (surge #2) when the number of COVID-19 patients on those units increased. These factors appeared to help health care workers overcome typical barriers to telehealth adoption and integrate the technology into their regular clinical routine [33,34].

Beyond the introduction of telehealth, the observed changes in nurse-patient encounters could also be due to unobserved differences between the primary COVID-19 unit and comparator units during this real-world health crisis. Aspects of nursing care were changing, in tandem with standards for the management of COVID-19, PPE availability, and infection control recommendations [32,35,36]. Telehealth encounters may have served as a replacement for nurse-to-patient calls on the bedside phone, which were not measured in this evaluation. Further, as the hospital was responding to the pandemic surges, fluctuations in patient acuity in all units impacted nurse-to-patient ratios. As the use of inpatient telehealth continues to evolve, evaluating the long-term use and sustainability outside of the pandemic setting may be an area for future research.

This retrospective, observational study utilized readily available data in a real-world setting and thus certain limitations exist. RTLS badges were worn consistently among nurses; however, limited compliance among other health care professionals such as physicians preclude analysis of other roles [9]. Only nurses with the role of “nurse” or “float nurse” who wore the RTLS badge were included; thus, health care workers identified under a different role (eg, certified nursing assistant) or nurses not wearing a badge (eg, broken badge) were not captured in this study. Further, since the RTLS sensors are linked to room numbers, RTLS-based badge data were merged with patient-level EHR data, which were limited to the presence of a patient in a patient room and their most recent COVID-19 status based on midnight census. Therefore, changes in patient census or location that occurred throughout the day were not captured. In addition, the telehealth platform captured all telehealth encounters that occurred within the four units. However, the purpose of the call, and the identity and role of participants (eg, nurse, patient, other health care worker, family) were not captured by the telehealth platform, nor were data on the quantity and duration of calls using the bedside phone captured. This eliminated the possibility of analyzing the purpose of the telehealth communication at the patient or nurse level, which is an area for future work.

Despite these limitations, an RTLS offers an alternative to other high-burden data sources of interest, such as ethnographic observation, to provide novel insights that may not otherwise be available. The strength of this evaluation is that movement data are available for nurses and linked with individual patient encounters in this novel clinical context.

### Conclusions

Assessment of nursing workflow change following the deployment of inpatient telehealth in the context of the COVID-9 pandemic was feasible utilizing RTLS data in combination with EHR data. Compared with those of other units with shared mobile health units, direct nurse-patient encounters on a COVID-19 unit with in-room ready access to telehealth decreased in frequency and increased in duration, leading to a redistribution of work that did not impact total time at the bedside relative to prepandemic periods. The simultaneous adoption of telehealth suggests virtual care complemented, rather than replaced, in-person care in this setting. Study limitations, including the lack of telehealth utilization data at the nurse or patient level and multiple differences between the COVID-19 and comparator units (ease of telehealth availability and proportion of COVID-19 patients), preclude our ability to draw a causal link between nursing workflow change and telehealth adoption. Further evaluation is needed to determine potential downstream implications on unmeasured outcomes such as disease transmission, PPE utilization, and patient safety.

## Appendix

Multimedia Appendix 1 Methodological details, including telemedicine deployment and time intervals.

Multimedia Appendix 2 Average inpatient census by COVID-19 status (Table S1).

## Abbreviations

EHR: electronic health record
PPE: personal protective equipment
RTLS: real-time locating system
