# Supplementary material for: Nursing Workflow Change in a COVID-19 Inpatient Unit Following the Deployment of Inpatient Telehealth: Observational Study Using a Real-Time Locating System
Source: J Med Internet Res. 2022 Jun 17;24(6):e36882. doi: 10.2196/36882 (PMC9208574; doi:10.2196/36882)
Supplement: Multimedia Appendix 1 [file jmir_v24i6e36882_app1.docx]

**Methodological Details Including Telemedicine Deployment, Time Intervals**

A1. Defining Surge and Non-Surge Time Intervals

To define surge and non-surge time intervals for the purpose of analysis, a surge period was defined as twice the state’s definition of “widespread” risk level of more than 7 new cases per day in 100,000 individuals.^1^ As the health system is situated between Santa Clara and San Mateo counties, daily cases for each of these counties^2^ were multiplied by 100,000 and divided by the 2019 overall population of each county projected from census data^3,4^ to define surge entry and exit dates. The midpoint between the dates for each county was then used in analysis.

For this evaluation, 6 unique study phases were identified: 1) Pre-pandemic [January 1, 2020 – March 14, 2020], 2) Initial pandemic response and telemedicine implementation [Telemedicine Roll-out; March 15, 2020 – April 15, 2020], 3) 1^st^ non-surge stage of the pandemic and telemedicine [Non-surge #1; April 16, 2020 – July 10, 2020], 4) 1^st^ local surge of pandemic and telemedicine [Surge #1; July 11, 2020 – September 7, 2020], 5) 2^nd^ non-surge of pandemic and telemedicine [Non-surge #2; September 8, 2020 – November 10, 2020], and 6) 2^nd^ local surge of pandemic and telemedicine [Surge #2; November 11, 2020 – December 27, 2020].

A2. Telemedicine Deployment

Using a hub and spoke model, hardware, computers with video capability or full-sized tablets (Apple, Cupertino, California, USA), mounted on carts were centrally located within units as the “hubs” and “spokes” were full-sized tablets for patient use. In the primary COVID-19 unit, a dedicated tablet was assigned to each patient room; whereas, other units had multiple, wheeled carts with tablets to be moved into patient rooms as needed. The chosen video conferencing software (Zoom, San Jose, California, USA) is HIPAA-compliant, met minimum audio and visual quality specifications, and allowed the “hub and spoke configuration” where “spoke” tablets in the patient rooms automatically answered calls from “hubs”. These units complemented pre-existing bedside telephones in which a patient could press a red button to call out to their nurse.

A3. Access to RTLS Data

Access and use of the RTLS data required sensitivity and approval from various stakeholders, including administration, human resources, and the local nurse union, as monitoring of staff location has privacy concerns.^5–7^ Anonymization of nurses and focus evaluation of nurse movement in to and out of patient rooms, as opposed to all locations, were applied here to address these concerns. Once access was achieved, however, the RTLS data was able to capture changes in the frequency and duration of in-person encounters between nurses and patients providing insight onto the potential benefits of telemedicine during a pandemic. Overall, RTLS data, where it exists, must be handled with sensitivity given understandable staff resistance to monitoring of their location; using the minimum amount of anonymized data should be considered with relevant stakeholder approvals.

**References**

1. Risk Tier Categories. COVID19.CA.GOV. Published 2020. Accessed February 8, 2021. https://covid19.ca.gov/

2. Daily Case Counts by County. COVID-19: California Case Statistics, drawn from CA.gov. Published February 2021. Accessed February 12, 2021. https://public.tableau.com/views/COVID-19CasesDashboard_15931020425010/Cases?:embed=y&:showVizHome=no

3. Santa Mateo County. Data Commons Place Explorer. Published 2020. Accessed February 12, 2021. https://datacommons.org/place/geoId/06081

4. Santa Clara County. Data Commons Place Explorer. Published 2020. Accessed February 12, 2021. https://datacommons.org/place/geoId/06085

5. Patel B, Vilendrer S, Kling SMR, et al. Using a Real-Time Locating System to Evaluate the Impact of Telemedicine in an Emergency Department During COVID-19: Observational Study. *J Med Internet Res*. 2021;23(7):e29240. doi:10.2196/29240

6. Ho HJ, Zhang ZX, Huang Z, Aung AH, Lim W-Y, Chow A. Use of a Real-Time Locating System for Contact Tracing of Health Care Workers During the COVID-19 Pandemic at an Infectious Disease Center in Singapore: Validation Study. *J Med Internet Res*. 2020;22(5):e19437. doi:10.2196/19437

7. Kamel Boulos MN, Berry G. Real-time locating systems (RTLS) in healthcare: a condensed primer. *Int J Health Geogr*. 2012;11:25. doi:10.1186/1476-072X-11-25
