# Supplementary material for: Nursing Workflow Change in a COVID-19 Inpatient Unit Following the Deployment of Inpatient Telehealth: Observational Study Using a Real-Time Locating System
Source: J Med Internet Res. 2022 Jun 17;24(6):e36882. doi: 10.2196/36882 (PMC9208574; doi:10.2196/36882)
Supplement: Multimedia Appendix 2 [file jmir_v24i6e36882_app2.docx]

**Average inpatient census by COVID-19 status**

The patient census by COVID-19 status on the intervention and comparator units in each stage of the pandemic are described below in **Table S1**. In the first 4 stages in the pandemic, the primary COVID-19 unit housed the majority of patients with COVID-19 whereas the comparison units rarely cared for COVID-19 units before Surge #2. The comparison units primarily served patients without COVID-19, but the primary COVID-19 unit also cared for patients without COVID-19.

**Table S1.** Average inpatient census by COVID-19 status on a primary COVID-19 unit and three comparison units during the SARS-CoV-2 pandemic

| **Time period** | **Shift** | **Primary COVID-19 Unit** | | **Comparison Units** | |
| --- | --- | --- | --- | --- | --- |
|  |  | **Patients with COVID-19 per shift**  **(mean (SD), range)** | **Patients without COVID-19 per shift**  **(mean (SD), range)** | **Patients with COVID-19 per unit per shift**  **(mean (SD), range)** | **Patients without COVID-19 per unit per shift**  **(mean (SD), range)** |
| Pre-pandemic and pre-telemedicine | AM | 0.0 (0.0), 0 - 0 | 18.7 (1.4), 14 - 20 | 0.0 (0.0), 0 - 0 | 18.1 (2.2), 8 - 20 |
|  | PM | 0.0 (0.0), 0 - 0 | 22.6 (1.9), 18 - 27 | 0.0 (0.0), 0 - 0 | 22.0 (2.8), 8 - 28 |
| Telemedicine deployment | AM | 3.8 (2.8), 0 - 9 | 4.4 (5.9), 0 - 18 | 0.0 (0.0), 0 - 0 | 14.3 (3.7), 4 - 20 |
|  | PM | 5.3 (3.6), 0 - 12 | 6.3 (7.6), 0 - 23 | 0.0 (0.0), 0 - 0 | 17.2 (4.3), 5 - 25 |
| Non-surge #1 | AM | 4.1 (2.1), 0 - 10 | 4.8 (3.5), 0 - 12 | <0.1 (0.1), 0 - 1 | 16.8 (2.5), 9 - 20 |
|  | PM | 5.4 (2.5), 1 - 14 | 6.7 (4.2), 0 - 15 | <0.1 (0.1), 0 - 1 | 19.9 (3.0), 13 - 27 |
| Surge #1 | AM | 10.6 (2.9), 3 - 16 | 2.7 (1.4), 0 - 6 | <0.1 (0.1), 0 - 1 | 18.7 (1.3), 14 - 20 |
|  | PM | 13.2 (3.1), 7 - 19 | 4.0 (1.9), 0 - 9 | <0.1 (0.2), 0 - 1 | 22.4 (2.1), 17 - 29 |
| Non-surge #2 | AM | 4.9 (2.2), 1 - 11 | 8.4 (3.1), 1 - 14 | 0.2 (0.6), 0 - 3 | 18.3 (1.6), 11 - 20 |
|  | PM | 6.7 (2.6), 1 - 13 | 11.3 (3.7), 2 - 18 | 0.2 (0.6), 0 - 3 | 21.8 (2.2), 14 - 26 |
| Surge #2 | AM | 2.9 (1.5), 0 - 7 | 10.6 (2.6), 6 - 16 | 0.4 (1.1), 0 - 6 | 17.5 (3.4), 5 - 20 |
|  | PM | 3.8 (2.0), 0 - 9 | 13.5 (2.6), 9 - 18 | 0.5 (1.4), 0 - 6 | 20.6 (4.1), 6 - 28 |
